# Inhibitory Effects of Heat-Processed *Gynostemma pentaphyllum* Extract (Actiponin^®^) and Its Components on Cartilage Breakdown in Osteoarthritis

**DOI:** 10.3390/ijms26041728

**Published:** 2025-02-18

**Authors:** Seul Ah Lee, Chan Hwi Lee, Sun Hee Lee, Eunju Do, Do Kyung Kim, Tae-Lin Huh, Chun Sung Kim

**Affiliations:** 1 Department of Oral Biochemistry, College of Dentistry, Chosun University, Gwangju 61452, Republic of Korea; seulah21@naver.com; 2 TG Biotech Research Institute, Technobuilding, Kyungpook National University, 47, Gyeongdae-ro 17-gil, Buk-gu, Daegu 41566, Republic of Korea; cksgnl0201@tgbio.com (C.H.L.); ihappy278@tgbio.com (S.H.L.); ejdo2302@tgbio.com (E.D.); 3 Department of Oral Biology, College of Dentistry, Chosun University, Gwangju 61452, Republic of Korea; kdk@chosun.ac.kr

**Keywords:** *Gynostemma pentaphyllum*, actiponin, osteoarthritis, chondrocytes, anti-arthritic

## Abstract

Osteoarthritis (OA), caused by the long-term use of joints, is a representative degenerative disease in the elderly. However, recently, the age of onset has been decreasing owing to excessive activities among young people in their 20s and 30s. *Gynostemma pentaphyllum* (Thunb.) Makino (GP), a perennial herb of the Cucurbitaceae family, has been used since the Ming dynasty as a medicinal material to treat various ailments, such as rheumatism, liver disease, and diabetes. In this study, we investigated the anti-arthritic effects of heat-processed *Gynostemma pentaphyllum* extract (Actiponin (AP)) and its derivatives, damulin A (DA) and damulin B (DB), using in vitro (primary rat chondrocytes and SW1353 cells) and in vivo (destabilization of the medial meniscus (DMM)-induced OA model) systems. Histological analysis results from the in vivo study showed that the group that underwent DMM surgery induced degeneration by the loss of proteoglycan and the destruction of cartilage (OARSI score 14 ± 0.57), whereas the group that received AP daily for 8 weeks maintained an intact condition (OARSI score 5 ± 0.28 at 200 mg/kg, *p* < 0.001). In addition, cartilage thickness and chondrocytes were reduced in the DMM group, but were restored in the AP-administered group. Furthermore, the von Frey analysis results showed that the pain threshold of the DMM group was considerably low (54.5 g at 8 weeks), whereas that of the AP group was dose-dependently increased (65.5, 69.5, 70.3, and 71.8 at 8 weeks for 30, 50, 100, and 200 mg/kg, respectively). In vitro studies showed that AP, DA, and DB reduced the expression of interleukin-1β alone-induced nitrite; inducible nitric oxide synthase; cyclooxygenase-2; matrix metallopeptidase 1/3/13; and a disintegrin and metalloproteinase with thrombospondin motifs 4/5. They also restored the expression of collagen type II and aggrecan, which are components of the extracellular matrix. The anti-arthritic effects of AP, DA, and DB were confirmed to be mediated by the mitogen-activated protein kinase and nuclear factor kappa-light-chain-enhancer of activated B cell signaling pathways. Collectively, these results suggest that AP is a potential therapeutic agent for mitigating OA progression and chondroprotection.

## 1. Introduction

Osteoarthritis (OA) is a natural degenerative disease that occurs with aging; however, recently, the age of onset has decreased owing to the increase in excessive exercise among young people in their 20s and 30s. OA is caused by multiple factors other than aging, including trauma, obesity, genetics, sex, and environmental factors. The main cause is inflammation within the articular cartilage [1,2]. The primary function of the articular cartilage is to provide a smooth, lubricated surface for the joint and facilitate load transfer with a low coefficient of friction [1]. Articular cartilage has no blood vessels or nerves; therefore, wear and tear of the cartilage go easily unnoticed, and its ability for self-repair is limited [1]. From this perspective, maintaining healthy articular cartilage is important for preventing OA. Articular cartilage is composed of ≈2% chondrocytes and an extracellular matrix consisting of water, collagen, and proteoglycans [1,3]. Chondrocytes sparsely distributed in the articular cartilage do not form cell–cell contacts and are responsible for the extracellular matrix (ECM) environment [1,3]. The ECM surrounds chondrocytes, protecting them from the impact of loads and maintaining the cartilage structure [1]. Therefore, maintaining the integrity of chondrocytes and the ECM is the most fundamental way to protect joint health.

Inflammation is thought to be the main cause of OA, and among the factors that induce inflammation in articular cartilage, interleukin (IL)-1β is a powerful driver that triggers OA. IL-1β stimulates chondrocytes to secrete matrix-degrading enzymes, depleting proteoglycans, a major component of the ECM, destroying the collagen network, and reducing the synthesis of cartilage matrix proteins [2,4]. In addition, IL-1β increases the expression of inflammation-related factors such as cyclooxygenase (COX)-2, prostaglandin E_2_ (PGE_2_), nitric oxide (NO), inducible nitric oxide synthase (iNOS), tumor necrosis factor-alpha (TNF-α), and IL-6, and also stimulates adjacent cells by acting as a paracrine agent [3,4]. The overexpression of IL-1β and an inflammatory environment disrupt chondrocyte homeostasis and promote the expression of cartilage matrix-degrading enzymes [5]. Matrix metalloproteinases (MMPs) can degrade all ECM components, particularly MMP-1 and MMP-13, which can degrade collagen type II and aggrecan, the major components of the ECM, further intensifying OA [6]. A disintegrins and metalloproteinases with thrombospondin domains (ADAMTSs) are extracellular proteinases that affect ECM synthesis and degradation [7,8]. ADAMTSs are enzymes that primarily degrade aggrecan in cartilage in OA [9]. This is accomplished by separating the G1 and G2 of aggrecan by cleaving the Glu^373^–Ala^374^ bond in the interglobular domain [10,11]. According to these stimuli and inflammatory responses, downstream signaling pathways are also abnormally stimulated, and one of these pathways is the nuclear factor kappa-light-chain-enhancer of the activated B cell (NF-κB) pathway and mitogen-activated protein kinase (MAPK), which plays an important role in immune and inflammatory responses [12,13]. The MAPK pathway is an important regulator of inflammatory cytokine activity in chondrocytes, and the phosphorylation of MAPKs (ERK, JNK, and p38) signaling also prompts the expression of MMPs, ADAMTSs, iNOS, COX-2, and IL-1β. These stimuli damage the cartilage and ECM [13].

OA can be treated surgically and medically; however, depending on the patient’s symptoms, surgical treatment may or may not be feasible. In addition, the costs and adverse effects are considerable. In addition, medical treatment is only 20% effective with nonsteroidal anti-inflammatory drugs (NSAIDs), even though pain is reduced. In addition, NSAIDs are ineffective, and side effects such as hospitalization, gastrointestinal disorders, and death owing to peptic ulcers occur in 10–20% of patients; safety cannot be guaranteed with long-term use [14,15,16]. Therefore, there is a need to develop more effective medicines with fewer side effects. Numerous researchers have developed traditional pharmaceutical agents and supplements derived from natural products. Among the natural products that have been shown to be effective against OA are methylsulfonylmethane (MSM), curcumin, and pycnogenol [17,18,19]. However, their effects are insignificant, and further research on their antioxidant effects, major components, and side effects is required to ensure safety. In addition, glucosamine and chondroitin are widely used to improve OA, but glucosamine is ineffective in some formulations compared with a placebo [15,17]. Glucosamine is associated with many risk factors owing to its poor clinical relevance and lack of research. Similarly to glucosamine, chondroitin is ineffective and has been understudied.

Therefore, research on active ingredients derived from natural products is both important and necessary. The use of medicinal plants to treat illnesses and to promote health is an age-old practice [20]. *Gynostemma pentaphyllum* (Thunb.) Makino (GP) is a perennial herb of the Cucurbitaceae family that has been used as a medicinal material to treat rheumatism, liver diseases, and diabetes [21]. GP has long been used in various Asian countries, including Korea, China, Japan, and Vietnam, as a traditional tea and herbal medicine [21,22]. It contains more than 20 active ingredients, including dammarane saponins, flavonoids, amino acids, and phytosterols and has been reported to have antioxidant, anticancer, hepatoprotective, anti-atherogenic, and hypoglycemic effects [21,22,23,24]. Gypenoside, a dammarane saponin, is well known as the main active ingredient responsible for the pharmacological effects of GP [21,25]. Recent studies have reported that gypenoside improves anxiety and depression through anti-inflammatory effects in the hippocampus and prefrontal cortex of anxious and depressed mice and effectively inhibits growth and glycolysis in gastric cancer by targeting the Hippo pathway [26,27]. In our previous study, we found that damulin A (DA) and damulin B (DB) were present in 10-fold higher quantities in heat-processed GP extract than in GP extract, and they were the main active ingredients responsible for its anti-obesity effect [28]. This previous study confirmed that DA and DB increased the phosphorylation of adenosine monophosphate-activated protein kinase (AMPK) and acetyl-CoA carboxylase (ACC) in HepG_2_ and L6 cells, thereby stimulating fat oxidation and glucose absorption, resulting in reduced fat mass [28,29]. The heat-processed GP extract, named Actiponin (AP), was approved as a functional ingredient for health functional foods by the Ministry of Food and Drug Safety of Korea in 2013. In our previous study, we confirmed that AP has anti-inflammatory and anti-obesity effects, and in this study, we aimed to analyze its anti-arthritic efficacy and mechanism based on those results, because obesity and the development of degenerative arthritis are very closely related [30].

## 2. Results

### 2.1. HPLC/MS of AP and Its Major Gypenosides

**Figure 1:**
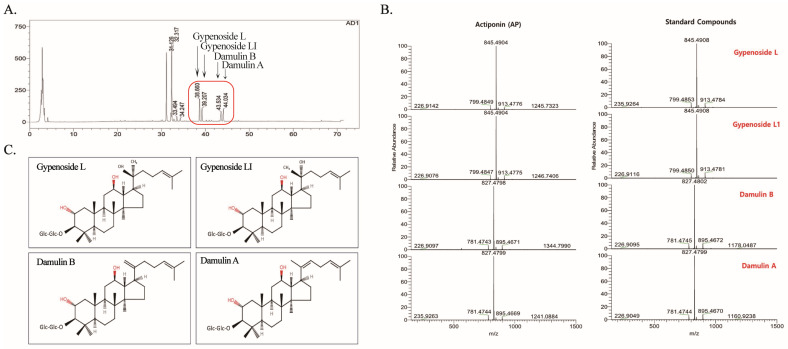
HPLC/MS analysis of Actiponin (AP). (**A**) HPLC chromatogram of Actiponin. Red rectangle represents major gypenosides present in AP. (**B**) Major gypenoside compounds identified by HPLC/MS analysis of AP and standard gypenoside compounds. (**C**) Chemical structure of gypenoside L, gypenoside LI, damulin B and damulin A.

To characterize the major components of AP, HPLC analysis was performed, according to our previous study [29]. The retention times of the biological major components of AP were 38.660 min for gypenoside L, 39.207 min for gypenoside LI, 43.534 min for DB, and 44.034 min for DA (Figure 1A). In particular, the HPLC/MS analysis of AP displayed the presence of the gypenoside L, gypenoside LI, DB, and DA at RT 6.51, 6.83, 9.69, and 9.99 min, and the peaks were confirmed by comparison with the HPLC/MS chromatogram of each of the standard compounds (Figure 1B). Figure 1C showed the structure of the four major components (gypenoside L, gypenoside LI, DB, and DA).

### 2.2. Effects of AP Administration in the Articular Cartilage of Destabilization of the Medial Meniscus (DMM) OA Rat Model

**Figure 2:**
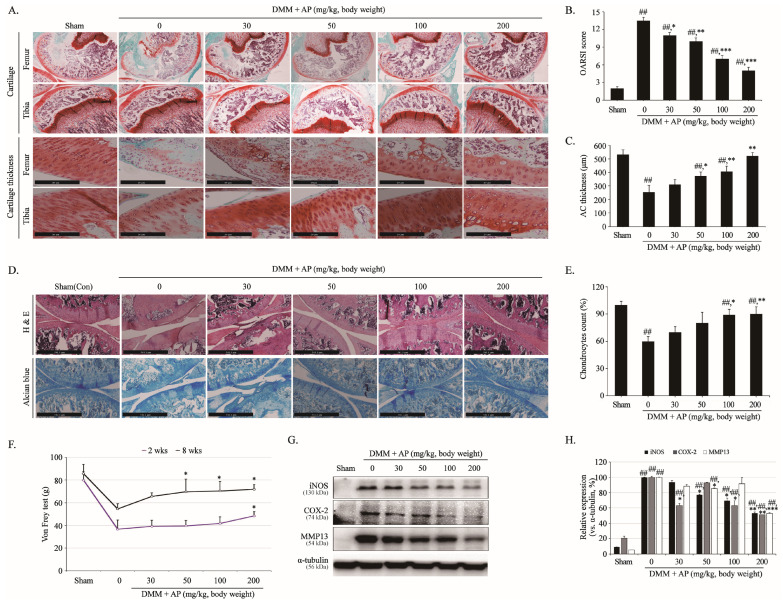
Histological evaluation of cartilage-protective effect of AP against cartilage degradation in DMM model. After sham or DMM surgery, rats received gavage of distilled water and AP every day for 8 weeks. Histological analysis of cartilage destruction was evaluated by safranin O/Fast Green, scale bar = 20 μm (**A**) and H&E and Alcian blue, scale bar = 541.1 μm (**D**). (**B**) Osteoarthritis Research Society International (OARSI) advanced Osteoarthritis Cartilage Histopathology Assessment System. (**C**) Articular cartilage (AC) thickness was evaluated by staining safranin O thickness using Leica application suite X program. (**E**) Number of chondrocytes in cartilage tissue was counted through a three-person blind test using H&E staining. (**F**) Measuring plantar pain using von Frey tests. (**G**) Protein levels of iNOS, COX-2, and MMP13 determined using Western blot. (**H**) Quantitative data of (**G**) were analyzed using ImageJ software (version 1.53). α-tubulin served as internal control. In vivo studies were performed three independent times, and Western blot analyses were performed at least five times. ANOVA and Dunnett tests were used to evaluate significance of results. ## *p* < 0.05 compared with sham group; * *p* < 0.05, ** *p* < 0.01, *** *p* < 0.001 compared with DMM group.

The in vivo chondroprotective effect of AP was confirmed using the DMM model, which is a surgical procedure used to induce OA through an incision of the medial meniscus and is most similar to the pathogenesis of OA in humans. To determine the chondroprotective effect, safranin O/Fast Green staining, hematoxylin and eosin (H&E) staining, and Alcian blue staining were performed. The von Frey test was performed to confirm pain reduction, and the expression of cartilage matrix-degrading enzymes in the tissue was analyzed using Western blot analysis. As a result of performing safranin O/Fast Green staining, which is a representative histological analysis of cartilage, weaker staining and rougher surfaces were observed in the DMM group compared to the sham group (Figure 2A). On the other hand, in the group that received AP orally every day for 8 weeks, the staining was darker, and the surface roughness was reduced (Figure 2A). The results in Figure 2B were evaluated using Osteoarthritis Research Society International (OARSI) scores through a three-person blind test for Figure 2A. The score in the DMM group was 14 ± 0.57, while the scores in the groups administered 30, 50, 100, and 200 mg/kg AP were 11 ± 0.56, 10 ± 0.57, 7 ± 0.57, and 5 ± 0.28, which were decreases of 19%, 26%, 49%, and 65%, respectively (Figure 2B). In addition, analysis of articular cartilage (AC) thickness stained with safranin O showed that the tibia and femur cartilage thickness increased in the AP-treated group compared with the DMM group (Figure 2C). Similarly to the results of safranin O staining, the results of H&E and alcian blue staining also showed that the cartilage surface gradually became smoother in the AP-treated group compared to the DMM group (Figure 2D). Furthermore, H&E staining showed that the chondrocyte survival rate was higher in the AP-treated group compared to the DMM group, but no significance was observed at 30 mg/kg (Figure 2E). Two weeks after AP administration, there was no significant difference in pain reduction between the groups; however, pain was relieved at 8 weeks of AP 50 mg/kg or higher (Figure 2F). To analyze the cause of these effects, the protein expression of cartilage matrix-degrading enzymes and inflammatory factors was examined. Compared with the control group, the protein expression of iNOS, COX-2, and MMP13 in the DMM group was significantly increased, but AP reduced their expression in a concentration-dependent manner (Figure 2G,H). These results indicate that AP alleviates OA by decreasing cartilage matrix-degrading factors, increasing cartilage amounts, and attenuating cartilage thickness degradation in vivo.

### 2.3. Effects of AP, DA, and DB on Viability of Primary Chondrocytes and SW1353 Cells

**Figure 3:**
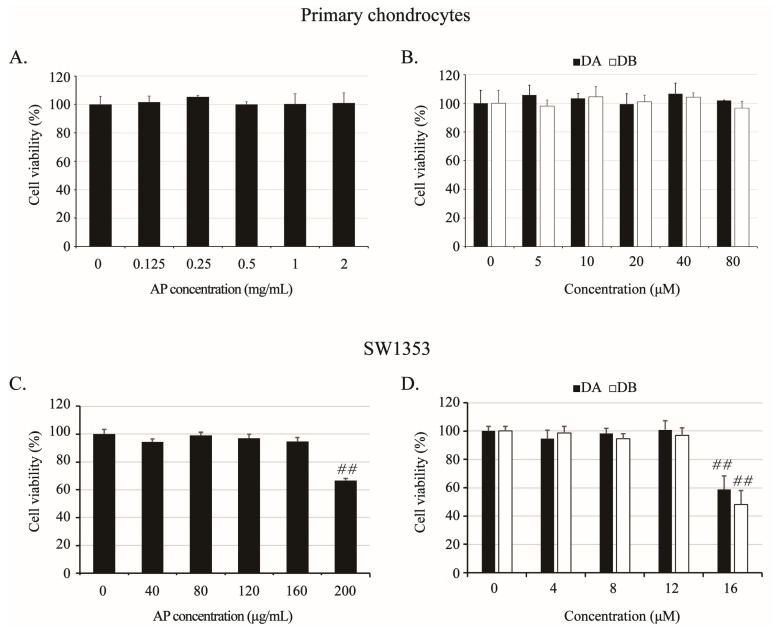
Effects of AP, DA, and DB on viability of rat primary chondrocytes and SW1353 cells. Cells were treated with AP (primary chondrocytes, 0.125, 0.25, 0.5, 1, and 2 mg/mL; SW1353, 40, 80, 120, 160, and 200 μg/mL), DA (primary chondrocytes, 5, 10, 20, 40, and 80 μM; SW1353, 4, 8, 12, and 16 μM), and DB (primary chondrocytes, 5, 10, 20, 40, and 80 μM; SW1353, 4, 8, 12, and 16 μM) for 24 h, and viability was determined by MTT assay. Cytotoxicity of AP (**A**), DA and DB (**B**) on primary chondrocytes; AP (**C**), DA and DB (**D**) on SW1353 cells. Untreated cells served as controls and were considered 100% viable. Data represented as mean ± SD of five independent experiments. ## *p* < 0.05 compared with control (0) group.

In all subsequent in vitro experiments, not only AP but also DA and DB, the active ingredients in AP’s anti-obesity effect, were tested together to evaluate whether they were effective ingredients in improving joints. The effects of AP, DA, and DB on cell viability were analyzed using the MTT assay, which demonstrated that AP had no effect on cell viability up to 2 mg/mL, whereas DA and DB had no effects up to 80 μM in primary chondrocytes (Figure 3A,B). Furthermore, in SW1353 cells, AP was not cytotoxic up to 160 μg/mL, and DA and DB were not cytotoxic up to 12 μM (Figure 3C,D). Therefore, to avoid confounding cytotoxicity and efficacy, all subsequent experiments were performed at limiting concentrations of 0.5 mg/mL AP and 40 μM DA and DB for primary cultured chondrocytes and 160 μg/mL AP and 12 μM DA and DB for SW1353.

### 2.4. Effects of AP, DA, and DB on NO, PGE_2_, iNOS, and COX-2 Expression in IL-1β-Stimulated Primary Chondrocytes

**Figure 4:**
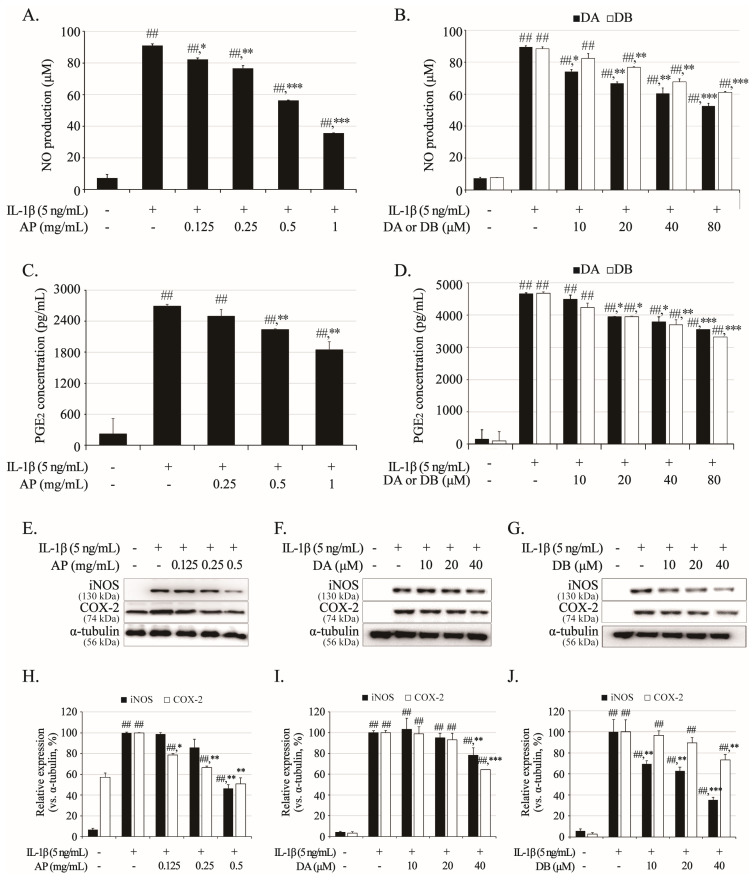
Inhibitory effects of AP, DA, and DB on IL-1β-induced nitrite, PGE_2_, iNOS, and COX-2. Primary chondrocytes were pre-treated with AP (0.125, 0.25, 0.5, and 1 mg/mL), DA (10, 20, 40, and 80 μM) and DB (5, 10, 20, 40, and 80 μM) for 1 h, followed by IL-1β (5 ng/mL) stimulation for 24 h. Nitrite production of AP (**A**), DA, and DB (**B**) was determined in cultured medium using Griess reagent. PGE_2_ production AP (**C**), DA and DB (**D**) was determined in cultured medium using ELISA kit. Expression of iNOS and COX-2 was determined using Western blot analysis; AP (**E**), DA (**F**) and DB (**G**). (**H**–**J**) Quantitative data of (**E**–**G**) were analyzed using ImageJ software (version 1.53). α-Tubulin served as internal control. *n* = 5 per group. Data are represented as mean ± SD of five independent experiments. ## *p* < 0.05 vs. control group; * *p* <0.05, ** *p* <0.01, and *** *p* < 0.001 compared with IL-1β-treated group.

Inflammatory factors such as NO, PGE_2_, iNOS, and COX-2 are the primary causes of OA exacerbation. IL-1β was used to induce inflammation in primary chondrocytes. Primary chondrocytes were seeded at 1 × 10^6^ cells/mL in 12-well cell culture plates and pretreated with AP, DA, and DB for 1 h, immediately followed by IL-1β (5 ng/mL) for 24 h. In the IL-1β-only-induced group, the expression levels of NO and PGE_2_ clearly increased (Figure 4A–D). AP pretreatment reduced the production of NO and PGE_2_ in a concentration-dependent manner, decreasing them by 61% and 32% at 1 mg/mL to 35.70 ± 0.09 μM and 1842.889 ± 157.232 pg/mL, respectively (Figure 4A,C). In addition, DA and DB, the active components of AP, significantly suppressed IL-1β-induced NO and PGE_2_ production in a concentration-dependent manner, similar to the efficacy of AP against NO and PGE_2_ (Figure 4B,D). Analysis of the up-regulators of NO and PGE_2_ showed that AP, DA, and DB significantly suppressed iNOS and COX-2 expression in a concentration-dependent manner (Figure 4E–J). These results indicate that AP, DA, and DB exert potential anti-inflammatory effects by inhibiting the inflammatory response of IL-1β.

### 2.5. Effects of AP, DA, and DB on Expression of Matrix-Degrading Enzymes in IL-1β-Stimulated Primary Chondrocytes and SW1353 Cells

**Figure 5:**
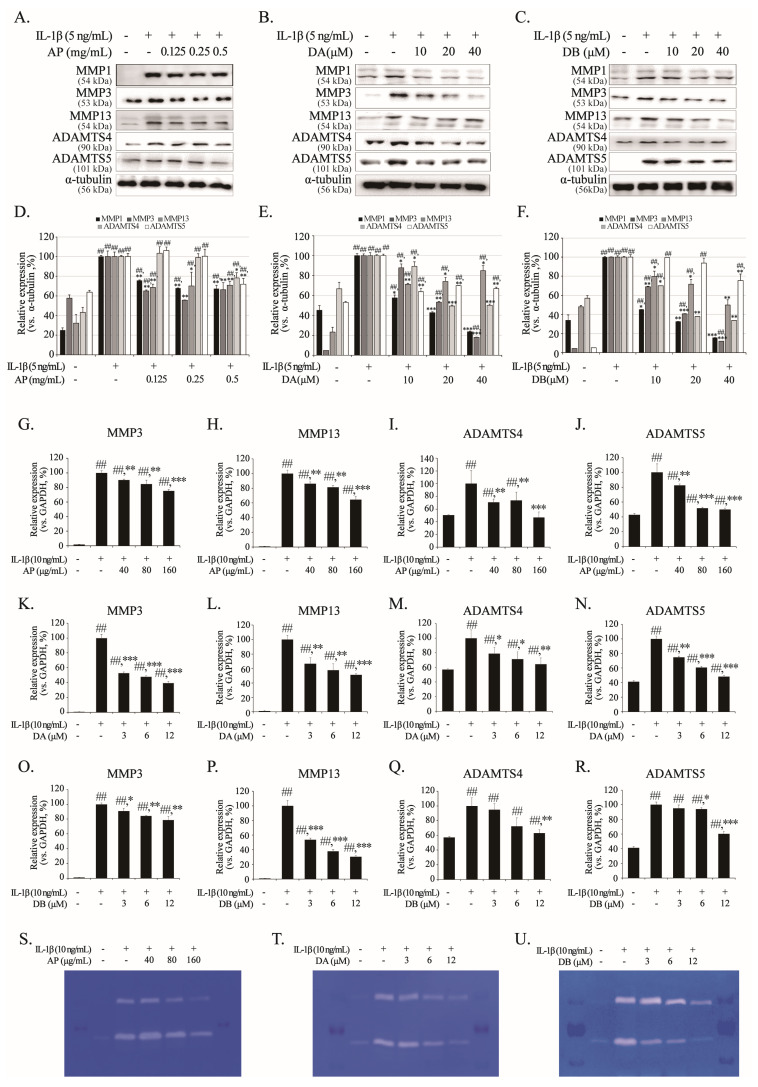
Inhibitory effects of AP, DA and DB on IL-1β-induced MMP1, MMP3, MMP13, ADAMTNS4, and ADAMTS5. Primary chondrocytes and SW1353 cells were pre-treated with AP, DA, and DB for 1 h, followed by IL-1β stimulation for 24 h. Protein levels of MMP1, MMP3, MMP13, ADAMTS-4, and ADAMTS5 in primary chondrocytes were determined using Western blot analysis; AP (**A**), DA (**B**), and DB (**C**). (**D**–**F**) Quantitative data of (**A**–**C**) were analyzed using ImageJ software (version 1.53). α-tubulin served as internal control. mRNA levels of MMP3, MMP13, ADAMTS4, and ADAMTS5 in SW1353 cells were determined using real-time PCR; AP (**G**–**J**), DA (**K**–**N**), and DB (**O**–**R**). Activities of secreted cartilage-degrading enzymes were analyzed using gelatin zymography; AP (**S**), DA (**T**) and DB (**U**). *n* = 5 per group. Data are represented as mean ± SD of five independent experiments. ## *p* < 0.05 vs. control group; * *p* < 0.05, ** *p* < 0.01, and *** *p* < 0.001 compared with the IL-1β-treated group.

NO and PGE_2_ act as inflammatory mediators, accelerating the activation of matrix-degrading enzymes such as MMPs and ADAMTSs, which degrade aggrecan and the ECM. Therefore, we analyzed the expression of MMP1/3/13 and ADAMTS4/5 in primary chondrocytes and their mRNA expression in SW1353. In the group treated with IL-1β alone, the protein expression of MMP1/3/13 and ADAMTS4/5 increased, whereas the expression of these proteins was significantly suppressed in the groups pretreated with AP, DA, and DB (Figure 5A–F). Analysis of the mRNA levels in SW1353 showed that, similar to the protein levels, AP, DA, and DB significantly suppressed the mRNA expression of MMP3/13 and ADAMTS4/5, which was increased by IL-1β (Figure 5G–R). In addition, the gelatin zymography results indicated that the expression level of MMP increased in the IL-1β-only-induced group (Figure 5S–U). These findings reveal that AP, DA, and DB inhibit cartilage-degrading enzymes in IL-1β-induced conditions.

### 2.6. Effects of AP, DA, and DB on Degradation of Aggrecan and Collagen Type II in IL-1β-Stimulated Primary Chondrocytes and SW1353 Cells

**Figure 6:**
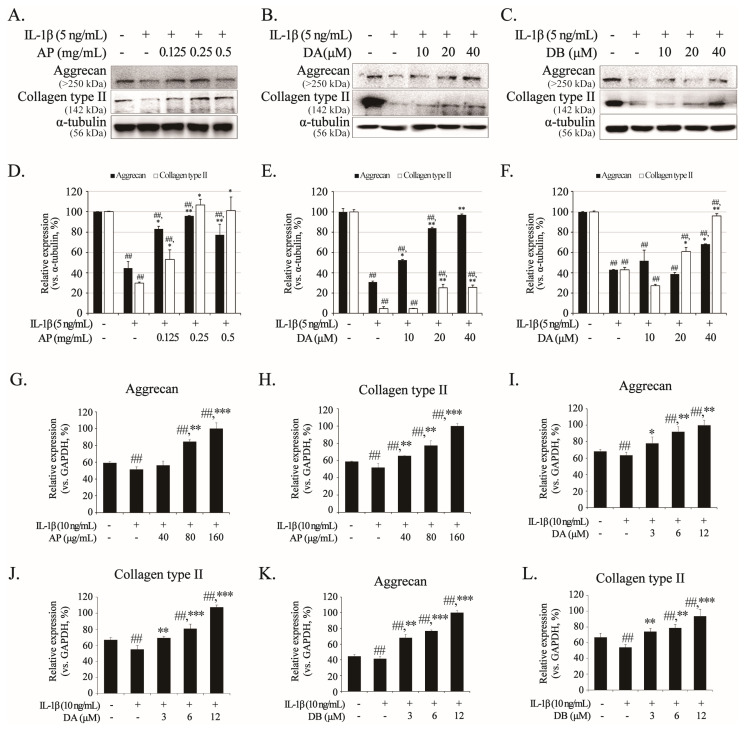
Inhibitory effects of AP, DA, and DB on IL-1β-induced aggrecan and collagen type II degradation. Primary chondrocytes and SW1353 cells were pre-treated with AP, DA and DB for 1 h, followed by IL-1β stimulation for 24 h. Protein levels of aggrecan and collagen type II in primary chondrocytes were determined using Western blot analysis; AP (**A**), DA (**B**), and DB (**C**). (**D**–**F**) Quantitative data of (**A**–**C**) were analyzed using ImageJ software (version 1.53). α-tubulin served as an internal control. mRNA levels of aggrecan and collagen type II in SW1353 cells were determined using real-time PCR; AP (**G**,**H**), DA (**I**,**J**), and DB (**K**,**L**). *n* = 5 per group. Data are represented as mean ± SD of five independent experiments. ## *p* < 0.05 vs. control group; * *p* < 0.05, ** *p* < 0.01, and *** *p* < 0.001 compared with the IL-1β-treated group.

Aggrecan and collagen type II, major components of the cartilage ECM, are degraded by IL-1β-induced inflammation. Therefore, we investigated the effects of AP, DA, and DB on the mRNA and protein expression levels of aggrecan and type-II collagen in primary chondrocytes and SW1353 cells. In the groups pretreated with AP, DA, and DB, the protein degradation of aggrecan and collagen type II was effectively prevented, whereas in the group induced only with IL-1β, the protein expression levels of aggrecan and collagen type II were reduced (Figure 6A–F). Similarly to the Western results, the mRNA expression of aggrecan and collagen type II was deceased by IL-1β, but pretreatment with AP, DA, and DB restored the reduced expression (Figure 6G–L). These results indicate that AP, DA, and DB play important roles in cartilage protection by inhibiting the degradation of aggrecan and collagen type II in the IL-1β-induced group.

### 2.7. Effects of AP, DA, and DB on MAPK and NF-κB Signaling Pathways in IL-1β-Stimulated Primary Chondrocytes

**Figure 7:**
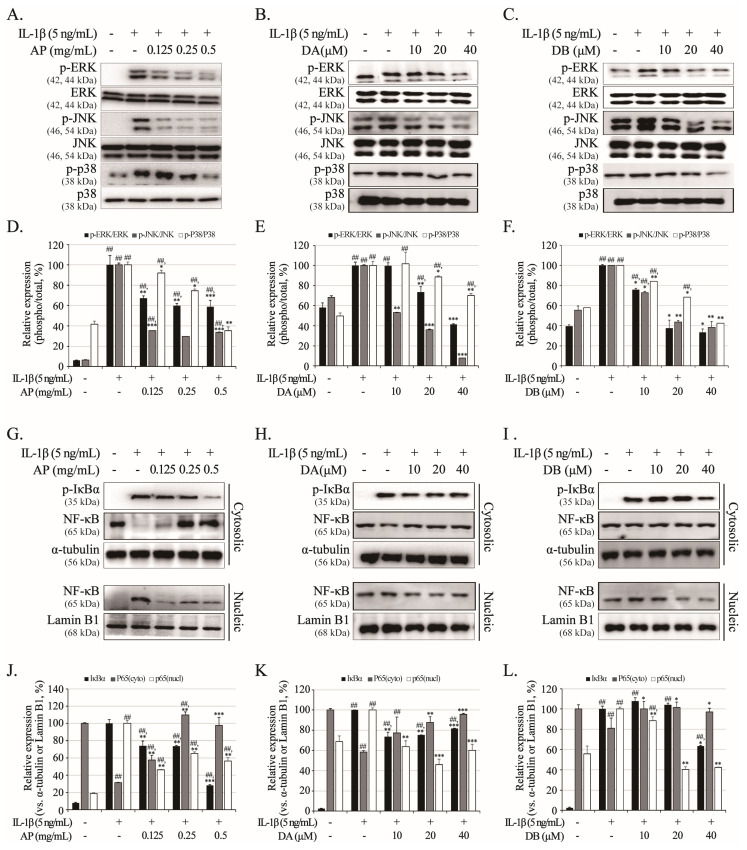
Effects of AP, DA, and DB on IL-1β-induced phosphorylation of MAPKs (ERK, JNK, and p38) and activation of NF-κB signaling. Primary chondrocytes were pre-treated with AP (0.125, 0.25, and 0.5 mg/mL), DA (10, 20, and 40 μM), and DB (10, 20, and 40 μM) for 1 h, followed by IL-1β (5 ng/mL) stimulation for 3 h. Protein expression levels of MAPK (ERK, JNK, and p38) and NF-κB (IκBα and p65) were determined using Western blot analysis. MAPKs: AP (**A**), DA (**B**), and DB (**C**); NF-κB: AP (**G**), DA (**H**), DB (**I**). (**D**–**F**,**J**–**L**) Quantitative data of (**A**–**C**,**G**–**I**) were analyzed using ImageJ software (version 1.53). *n* = 5 per group. Data are represented as mean ± SD of five independent experiments. ## *p* < 0.05 vs. control group; * *p* < 0.05, ** *p* < 0.01, and *** *p* < 0.001 compared with IL-1β-treated group.

The IL-1β-induced inflammatory response stimulates MAPK and NF-κB signaling, important signaling factors that regulate the transcription of cartilage-degrading enzymes such as MMPs, the ADAMTS family, and inflammatory mediators. Thus, the efficacy of AP, DA, and DB on MPAK and NF-κB signaling was examined in IL-1β-induced primary chondrocytes. In Figure 7A–F, IL-1β treatment for 3 h phosphorylated the MAPKs (ERK, JNK, and p38). However, pretreatment with AP, DA, and DB for 3 h, and with IL-1β, suppressed phosphorylation MAPKs (ERK, JNK, and p38) (Figure 7A–F). These results imply that MAPK activity can be modulated by AP, DA, and DB. In addition, IL-1β induced the nuclear translocation of p65 by inducing the phosphorylation of IκB-α, but pretreatment with AP, DA and DB prevented the nuclear translocation of NF-κB subunit p65 by inhibiting the phosphorylation of IκB-α (Figure 7G–L).

## 3. Discussion

OA is a common disease in the elderly, where cartilage wear is caused by continued usage and inflammation [6]. Because there is no practical treatment for OA, it is considered a significant social and economic burden. Moreover, cartilage damage is accelerated by damage to the cartilage matrix owing to the inflammatory response caused by tearing between cartilaginous tissues [1,6]. Inflammation strongly influences the pathogenesis of OA in response to cartilage catabolism, and inflammation-induced chondrocyte apoptosis impedes tissue maintenance and function [1,9]. In particular, IL-1β secreted as a result of inflammation promotes the release of cartilage-degrading enzymes such as MMPs, ADAMTS family members, and other catabolic enzymes and cartilage matrix degradation factors [3]. In addition, several studies have shown that the inhibition of IL-1β-induced inflammation mediators such as NO and PGE_2_ alleviates OA pathogenesis, pain, inflammation, and proteoglycan loss [3,5,7]. Therefore, the prevention of inflammatory mediators is of great importance in the treatment of OA. A key aspect of OA remission is the protection of chondrocytes from external stimuli and the prevention of ECM degradation. Representative over-the-counter medications for OA include acetaminophen, duloxetine, and nonsteroidal anti-inflammatory drugs (NSAIDs), which relieve inflammation and reduce pain [15,31]. However, when used for a long time, side effects such as gastrointestinal disorders and kidney toxicity can occur [32,33]. Therefore, it is crucial to develop effective treatments for OA with fewer side effects and high efficacy.

For this reason, we focused on *Gynostemma pentaphyllum* (GP) extract (i.e., AP). In our previous studies, we demonstrated that obesity is alleviated through AMPK activity and that this effect is mediated by DA and DB [28,29]. The anti-obesity efficacy of AP in in vitro and in vivo models was examined in a randomized, double-blind, placebo-controlled trial in obese subjects, in which supplementation for 12 weeks significantly reduced the total abdominal fat area and body weight without any significant side effects [34]. Despite these benefits, research on its anti-arthritic effects and underlying mechanisms is lacking. This study evaluated the anti-arthritic efficacy of AP in IL-1β-stimulated primary chondrocytes, SW1353 cells, and a DMM-induced OA model.

MMPs are proteolytic enzymes that are overexpressed in cartilage tissues and synovial fluid and degrade proteoglycans and collagen type II, which are components of the ECM that protect articular cartilage and chondrocytes in patients with OA [5,9]. In particular, MMP3 and MMP13 disrupt aggrecan and type II collagen, creating an appropriate microenvironment that exacerbates OA. Aggrecan degradation causes the release of sulfated glycosaminoglycans (sGAGs), which play an important role in cartilage elasticity by attaching to aggrecan core proteins to form proteoglycans [10,11]. ADAMTSs are the primary enzymes responsible for the degradation of the proteoglycan aggrecan during OA pathogenesis [8]. In our study, AP effectively decreased the expression levels of MMP1/3/13 and ADAMTS4/5 in IL-1β-induced rat primary chondrocytes and SW1353. Furthermore, AP, DA, and DB significantly inhibited the degradation of aggrecan and collagen type II, indicating that AP, DA, and DB effectively inhibited the increase in IL-1β-induced sGAG content.

IL-1β influences the initiation and development of OA and modulates the expression levels of MMPs, iNOS, and COX-2 through MAPKs and NF-κB signaling associated with inflammatory responses [12,13]. The MAPK signaling is achieved through multifunctional transducers of extracellular signals that activate various downstream pathways that regulate cellular physiological activities, including cell proliferation, death, survival, inflammation, and differentiation [35]. The blockading of the MAPK pathways reduces the production of inflammatory cytokines, and is therefore considered to be a very important signaling pathway in inflammatory responses [35,36]. The MAPK pathway is down-regulated by a negative feedback loop under conditions of excessive activity, regulating the inflammatory/immune regulatory balance and preventing chronic inflammation. Inflammatory factors activate transforming growth factor-β-activated kinase-1 (TAK1) through MYD88-dependent TLR4, and activated TAK1 induces the activation of mitogen-activated protein kinase (MAPK) and IKK complexes [36,37]. Activated IKK induces the activity of NF-κB, which includes phosphorylating and degrading IκB-α, and p65 bound to the cytoplasm by IκB-α is translocated to the nucleus to promote the transcription of inflammatory factors [37]. In our study, the phosphorylation of MAPKs (ERK, JNK, and p38) and NF-κB p65 by IL-1β treatment was significantly reduced in the AP-pretreated group in a dose-dependent manner.

In vivo, the anti-arthritic effect of AP was observed in a rat model of DMM surgery-induced OA. This model is widely used in OA pathogenesis associated with human aging through meniscal tears and degeneration [38,39]. Changes in chondrocyte and matrix components were confirmed through histological experiments, and cartilage destruction was observed in the DMM-surgery-induced OA rat model. However, in the group administered AP daily for 8 weeks, cartilage destruction was protected in the OA rat model, and the results were indicated by OARSI score. In addition, AP restored the number of chondrocytes and the thickness of cartilage cells, which were reduced owing to degeneration. Furthermore, as OA limits movement owing to pain, we analyzed whether AP could suppress pain. The results of the von Frey test showed that pain thresholds were significantly lower in the DMM group, but significantly higher in the AP-treated group. In vivo studies have demonstrated significant anti-osteoarthritis effects from 50 mg/kg, which corresponds to approximately 480 mg/kg in humans. Compared to other individually certified health functional food materials, 480 mg for humans is not a high intake, and since it is similar to the concentration currently on sale, it is considered very positive in terms of cost-effectiveness.

Collectively, these results suggest that AP exerts anti-arthritic effects. However, several issues remain unresolved. First, OA experiments should be conducted in large and small animals with long-term administration. Second, primary chondrocytes were isolated from young rats, an experimental method similar to that used in many other studies. However, OA usually occurs in older people; thus, it is necessary to conduct experiments using cells from old animals or patients with OA. Finally, AP, DA, and DB did not increased the protein expression of aggrecan and collagen type II, but increased the expression of mRNA. This is thought to be a necessary area to analyze to further clarify the anti-osteoarthritic mechanism of AP, DA, and DB, and research on the mechanism of AP related to increased mRNA synthesis is necessary. If these points are addressed, AP may be a potential candidate for the treatment of OA.

## 4. Materials and Methods

### 4.1. Reagents

Sulfanilamide, *N*-(1-naphthyl) ethylenediamine dihydrochloride, 3-(4,5-dimethylthiazol-2-yl)-2,5-diphenyltetrazolium bromide (MTT), and phosphoric acid were purchased from Sigma (St. Louis, MO, USA). IL-1β was purchased from ProSpec Protein Specialists (Rehovot, Israel). The PGE_2_ ELISA kit was purchased from R&D Systems (Minneapolis, MN, USA). Dulbecco’s modified Eagle’s medium/nutrient mixture F-12 (DMEM/F12) and penicillin–streptomycin solution were purchased from WelGene (Daegu, Gyeongsang-do, Republic of Korea). Fetal bovine serum (FBS) was purchased from ATLAS Biologicals (Fort Collins, CO, USA). Primary and secondary antibodies for MMP-13, ADAMTS-4, and iNOS (Abcam, Cambridge, MA, USA); anti-α-tubulin (Thermo Fisher Scientific, Waltham, MA, USA); MMP-1 (Lifespan Biosciences, Seattle, WA, USA), COX-2, TNF-α, and MMP-3 (Cell Signaling Technology, Danvers, MA, USA); NF-κB, IκB-α, and p-IκB-α (Invitrogen, Carlsbad, Germany); and Lamin B1 (Santa Cruz Biotechnology, Inc., Dallas, TX, USA) were also purchased. Gypenoside L, gypenoside LI, DA and DB were purchased from MedChemExpress (Monmouth Junction, NJ, USA).

### 4.2. Preparation of GP Extract (Actiponin ^®^), DA, and DB

Actiponin^®^ (AP), DA, and DB extracts were prepared according to the method de-scribed by Nguyen et al. [28,34,35]. To prepare AP, dried GP leaves (5 kg) were washed and subsequently underwent extraction with 8 volumes (*v*/*w*) of 50% ethanol, twice. The extracts were pooled and subjected to heating under high pressure and temperature. After 2.5 to 4.0 h of heating, the extract concentrate was spray-dried with maltodextrin to form a fine powder, and packaged. DA and DB were purified from the heat-treated extract of GP leaves by HP-20 ion exchange resin column chromatography (20 × 65 cm) as previously described [28], and the pure compounds were analyzed by HPLC (Shimadzu (CBM-40) System, Shimadzu, Kyoto, Japan).

### 4.3. High Performance Liquid Chromatography (HPLC) Analysis

The sample was analyzed using a Shimadzu HPLC system (Shimadzu, Kyoto, Japan) included a CBM-40 system controller, LC-40D pump, a SIL-40 autosampler, a CTO-40C column oven, and the detector was an Alltech 3300HP evaporative light scattering detector (ELSD, Deerfield, IL, USA). The sample solutions (2 mg/mL) were separated using a BDS Hyperil^TM^ C18 (4.6 mm i.d. × 250 mm, 5 μm; Thermo Fisher Scientific) at 40 °C. The injection volume was 10 μL. The mobile phase consisted of distilled water (A) and acetonitrile (B) at a flow rate of 0.9 mL/min. The gradient elution program used was as follows: 95% A (0–5 min), 95–27% A (5–45 min), 27–0% A (45–55 min), 0–0% A (55–60 min), 0–95% A (60–60.1 min), 95% (60.10–75 min).

### 4.4. Ultra-High-Pressure Liquid Chromatography (UHPLC)-HR MS Analysis of AP

LC/MS analyses were carried out using an LTQ Orbitrap XL (Thermo Electron Co., USA) coupled to an Accelar ultra-high-pressure liquid chromatography system (Thermo Electron Co.). Chromatographic separation of AP was conducted using an ACQUITY UPLC^®^ BEH C18 column (2.1 × 150 mm, 1.7 μm), operated at 40 °C and using mobile phases A (water with 0.1% formic acid) and B (acetonitrile with 0.1% formic acid). The solvent gradient conditions were with a flow rate of 0.4 mL/min and a gradient program of 40–80% B for 0–20 min. Each compound was detected with photodiode array at 200~600 nm. The MS analysis was performed with polarity switching, and the following parameters for MS/MS scans: *m*/*z* range of 150–1500; collision-induced dissociation energy of 45%; data-dependent scan mode. High-resolution mass spectra were acquired on an LTQ Orbitrap XL and analyzed with XCALIBUR software 4.1 (Thermo Fisher Scientific). UHPLC-MS analysis was supported by the Gyeonggido Business & Science Accelerator (GBSA, Gyeonggido, Republic of Korea).

### 4.5. Primary Rat Chondrocyte Isolation and Cell Viability Assay

Chondrocytes were isolated from the tibial plateau and femoral condyle of 5-day-old Sprague Dawley (SD) rats, as described by Kim et al. [29]. The SD rats sacrificed to isolate chondrocytes in this experiment were purchased from Damool Science (Daejeon, Republic of Korea), and articular cartilage was enzymatically digested using 0.3% (*w*/*v*) collagenase type II dissolved in DMEM/F12 at 37 °C in an incubator overnight. The digested cells and debris were filtered through a cell strainer (0.45 μm). The isolated chondrocytes were seeded at 1 × 10^6^ cells/mL in 6- and 12-well cell culture plates and cultured in a humidified incubator at 37 °C with 5% CO_2_ and DMEM/F12 containing 10% FBS and 1% penicillin/streptomycin. For cell viability, the chondrocytes were cultured up to 90% confluency and were treated with AP (primary chondrocytes: 0, 0.125, 0.25, 0.5, 1, and 2 mg/mL; SW1353: 40, 80, and 160 μM), DA, and DB (primary chondrocytes: 0, 5, 10, 20, 40, and 80 μg/mL; SW1353: 3, 6, and 12 μΜ) for 24 h. After 24 h, chondrocytes pretreated with AP, DA, and DB were added in a dose-dependent manner to MTT solution (5 mg/mL) in each well (100 μL/well), and the cells were incubated for 2 h at 37 °C. Two hours later, the cell culture medium containing the MTT solution was removed, and dimethyl sulfoxide (1 mL/well) was added to each well. The absorbance was measured at 595 nm. In addition, the primary rat chondrocytes were not passaged once during the experiments. All animal procedures and management protocols were approved by the Chosun University Institutional Animal Care and Use Committee (CIACUC2024-A0016).

### 4.6. Measurement of NO and PGE_2_ Production

Primary rat chondrocytes were seeded at 1 × 10^6^ cells/mL in 12-well cell culture plates and pretreated with AP, DA, and DB for 1 h, immediately followed by IL-1β (5 ng/mL) for 24 h. The accumulation of nitrite in the culture medium was measured to confirm NO production. Briefly, a mixture of culture medium (100 μL) with 100 μL of Griess’ reagent (1% sulfanilamide in 5% phosphoric acid, and 0.1% naphthylamide in H_2_O) were measured at 540 nm using a microplate reader (Epoch BioTek Instruments Inc., Winooski, VT, USA). PGE_2_ production was measured using the Parameter PGE_2_ Assay Kit according to the manufacturer’s protocol.

### 4.7. Total RNA Isolation and Real-Time Polymerase Chain Reaction (PCR)

RNA was isolated using the TRIzol reagent (Invitrogen, Thermo Fisher Scientific Inc.), following the manufacturer’s protocol. The ReverTra Ace qPCR RT Master Mix (TOYOBO, Osaka, Japan) was used to synthesize the cDNA, which was subjected to real-time PCR using the Luna Universal qPCR Master Mix (NEB, Ipswich, MA, USA). The results were calculated using the 2^−ΔΔCt^ method, with normalization to GAPDH, to determine the fold-change in data analysis. The primer sequences are shown in Table 1.

### 4.8. Western Blotting Analysis

Primary rat chondrocytes were pretreated with AP, DA, and DB for 1 h, and then IL-1β (5 ng/mL) was induced intracellularly for 3 h or 24 h without removing AP, DA, and DB. Cells were lysed with PRO-PREP protein-extraction solution (iNtRON Biotechnology, Seongnam-si, Republic of Korea), and total protein was isolated on ice for 30 min. In addition, cytoplasmic and nuclear proteins were isolated using NE-PER Nuclear and Cytoplasmic Extraction Reagent (Thermo Fisher Scientific), according to the manufacturer’s instructions. The dissolved protein was centrifuged at 4 °C and 14,000× *g* for 15 min, and the protein concentration was measured using a BCA protein assay kit (Pierce, Rockford, IL, USA). Qualified lysate protein (10 or 20 μg) was separated on 6%, 8%, 10%, or 12% sodium dodecyl sulfate polyacrylamide (SDS) gels. Proteins in the SDS gels were transferred onto polyvinylidene difluoride membranes (Bio-Rad Laboratories, Hercules, CA, USA). The membranes were blocked with 5% BSA to avoid the detection of non-specific proteins in 1× Tris-buffered saline containing 0.1% Tween 20 (1× TBST) at room temperature for 1 h, and incubated with primary antibodies (1:1000) at 4 °C overnight. The membrane was washed thrice with 1X TBST, and horseradish peroxidase (HRP)-conjugated secondary antibody diluted to 1:5000–10,000 was added and incubated for 1 h at room temperature. Protein bands were developed using an enhanced chemiluminescence (ECL) kit (Millipore, Bedford, MA, USA) and visualized using a MicroChemi 4.2 imager (DNR Bioimaging Systems, Jerusalem, Israel).

### 4.9. Gelatin Zymography

SW1353 cells were pretreated with AP, DA, and DB for 4 h and then induced with IL-1β (10 ng/mL) for 24 h. The supernatants of the cultured media (30 μL) were mixed with non-reducing sample buffer (375 mM Tris-HCl [pH 6.8], 50% [*v*/*v*] glycerol, 6% [*w*/*v*] SDS, 0.08% [*w*/*v*] bromophenol blue) and loaded on 10% SDS-PAGE containing 0.1% gelatin. After electrophoresis, the gels were placed with renaturation buffer (2.5% (*v*/*v*) Triton X-100, 50 mM Tris-HCl [pH 7.5], 5 mM CaCl_2_, 2 μM ZnCl_2_) with gentle shaking for 20 min, twice. The gels were incubated with zymogram incubation buffer (0.05% (*v*/*v*) Triton X-100, 50 mM Tris-HCl [pH 7.5], 0.2 M NaCl, 5 mM CaCl_2_, 2 μM ZnCl_2_) at 37 °C for 24 h. Then, the gels were stained with 0.1% (*w*/*v*) Coomassie Brilliant Blue R-250 for 30 min at room temperature and destained until clear bands were visible, followed by imaging with an Azure 300 (Azure Biosystems, Dublin, CA, USA).

### 4.10. DMM-Induced OA Model in Rats

Male SD rats (8 weeks old) weighing 300 g were purchased from Damool Science (Daejeon, Republic of Korea), with four rats per group. The animals were housed in a controlled environment (temperature: 21 ± 1 °C; humidity: 55 ± 5%; 12 h light/dark cycle), allowing free access to water and commercial pellet chow ad libitum. All animals were cared for according to the National Institutes of Health Guide and Use of Laboratory Animals [30].

The surgical model of DMM was a representative OA model commonly used in many laboratories. The SD rats were acclimatized for 1 week prior to the experiment. The rats were randomly divided into the following seven groups of four rats each: Group 1 (normal), Group 2 (Sham, 0.9% saline), Group 3 (DMM, 0.9% saline), and Groups 4–7 (30, 50, 100, and 200 mg/kg AP); each drug was orally administered as was. The DMM surgery was approved by the Chosun University Institutional Animal Care and Use Committee (CIACUC2024-A0016). The DMM surgical method is to incise the medial meniscal ligament (MMTL) above the left and right knees, which is performed after anesthetizing the rats with 2.5% isoflurane [18,31]. This method was performed for all groups except for the normal and sham groups. Rats in the sham group were not transected, but their skin was incised. Starting 1 day after DMM surgery, Groups 4–7 were orally administered AP daily for 8 weeks. Groups 2 and 3 were administered saline orally for the same period. All SD rats were euthanized on the same day by injecting of 60% carbon dioxide.

### 4.11. Von Frey Analysis

To analyze pain threshold, experimental animals were stimulated in the mid-plantar region using a von Frey apparatus. Measurements were taken at 2 and 8 weeks, with each measurement performed thrice, and the average values for the three measurements were plotted on the graph.

### 4.12. Histological Analysis and Staining

Articular cartilages were fixed in 10% neutral-buffered formalin, and then rinsed with 1×X PBS for 1 d. Washed articular cartilages were decalcified with 0.5 M EDTA (pH 7.4), and then the articular cartilages were dehydrated through a series of ethanol solutions and embedded in paraffin blocks. Lateral serial sections were sliced into 4 µm segments and stained with safranin O/Fast Green, H&E, and Alcian blue. Stained sections were photographed digitally using an EVOS Core microscope (Thermo Fisher Scientific) and Leica application suite × program (Leica, Wetzlar, Germany). The stained articular cartilage was scored according to the OARSI Advanced Osteoarthritis Cartilage Histopathology Assessment System (0–6.5). The degree of articular cartilage destruction was summed using the OARSI score [32].

### 4.13. Statistical Analysis

All data were obtained from independent experiments (except for in vivo experiments). The results are represented as mean ± standard deviation (SD). One-way analysis of variance (ANOVA) using Dunnett’s test was applied for multiple comparisons using GraphPad Prism software (version 5.0; GraphPad Software Inc., San Diego, CA, USA). Statistical significance was set at ### *p* < 0.005 compared with the control group and * *p* < 0.5, ** *p* < 0.05, *** *p* < 0.005 vs. the IL-1β- induced group.

## Figures and Tables

**Table 1:** Primer sequences for real-time PCR.

Primers	Sequences
GAPDH F	ATCTCTGCCCCCTCTGCTGA
GAPDH R	GCTAAGCAGTTGGTGGTGC
MMP3 F	CACTCACAGACCTGACTCGGTT
MMP3 R	AAGCAGGATCACAGTTGGCTGG
MMP13 F	CCAACCCTAAACATCCAAAAAC
MMP13 R	AAAAACAGCTCCGCATCAAC
ADAMTS4 F	TGCCCGCTTCATCACTGA
ADAMTS4 R	CAATGGAGCCTCTGGTTTGTC
ADAMTS5 F	TATGACAAGTGCGGAGTATG
ADAMTS5 R	TTCAGGGCTAAATAGGCAGT
Aggrecan F	GAAAGGCATCGTGTTCCATT
Aggrecan R	ACGTCCTCACACCAGGAAAC
Collagen type II F	CCTGAGTGGAAGAGTGGAGA
Collagen type II R	TCCATAGCTGAAATGGAAGC

## Data Availability

All data generated or analyzed during this study are included in this manuscript and its information files.

## Abbreviations

ADAMTS, a disintegrin and metalloproteinase with thrombospondin motifis; AP, Actiponin; COX-2, Cyclooxygenase-2; DA, damulin A; DB, damulin B; DMEM/F12, Dulbecco’s modified Eagle’s medium/nutrient mixture-F12; DMM, destabilization of the medial meniscus; ECL, enhanced chemiluminescence; ECM, extracellular matrix; ESLD, evaporative laser scattering detector; FBS, fetal bovine serum; GBSA, Gyeonggido Business & Science Accelerator; GP, *Gynostemma pentaphyllum*; H&E, hematoxylin and eosin; HPLC, high-performance liquid chromatography; HRP, horseradish peroxidase; IL-1β, interleukin-1beta; iNOS, inducible nitric oxide; MAPK, mitogen-activated protein kinase; MMP, matrix metallopeptidase; MMTL, medial meniscotibial ligament; MS, mass spectrometry; MSM, methyl sulfonylmethane; NF-κB, nuclear factor kappa-light-chain-enhancer of activated B cells; NO, nitric oxide; NSAIDs, nonsteroidal anti-inflammatory drugs; OA, osteoarthritis; OARSI, Osteoarthritis Research Society International; PGE_2_, prostaglandin E_2_; SDS, sodium dodecyl sulfate; sGAG, sulphated glycosaminoglycan; TNF-α, tumor necrosis factor-alpha.

## References

[1] Chen J., Shen W., Zhao T., Wang L., Han J.L., Hamilton H.J., Im H.J. (2017). Osteoarthritis: Toward a comprehensive understanding of pathological mechanism. Bone Res..

[2] Krakowski P., Rejniak A., Sobczyk J., Karpiński R. (2024). Cartilage integrity: A review of mechanical and frictional properties and repair approaches in osteoarthritis. Healthcare.

[3] Jenei-Lanzl Z., Meurer A., Zaucke F. (2019). Interleukin-1β signaling in osteoarthritis-chondrocytes in focus. Cell Signal..

[4] Hunziker E.B., Quinn T.M., Häuselmann H.J. (2002). Quantitative structural organization of normal adult human articular cartilage. Osteoarthr. Cartil..

[5] Maldonado M., Nam J. (2013). The role of changes in extracellular matrix of cartilage in the presence of inflammation on the pathology of osteoarthritis. Biomed. Res. Int..

[6] Hunter D.J., Bierma-Zeinstra S. (2019). Osteoarthritis. Lancet.

[7] Jang G., Lee S.A., Hong J.H., Park B.R., Kim D.K., Kim C.S. (2022). Chondroprotective Effects of 4,5-Dicaffeoylquinic Acid in Osteoarthritis through NF-κB Signaling Inhibition. Antioxidants.

[8] Majumdar M.K., Askew R., Schelling S., Stedman N., Blanchet T., Hopkins B., Morris E.A., Glasson S.S. (2007). Double-knockout of ADAMTS-4 and ADAMTS-5 in mice results in physiologically normal animals and prevents the progression of osteoarthritis. Arthritis Rheum..

[9] Knudson C.B., Knudson W. (2001). Cartilage proteoglycans. Semin. Cell Dev. Biol..

[10] Mercuri F.A., Doege K.J., Arner E.C., Pratta M.A., Last K., Fosang A.J. (1999). Recombinant human aggrecan G1-G2 exhibits native binding properties and substrate specificity for matrix metalloproteinases and aggrecanase. J. Biol. Chem..

[11] Aspberg A. (2012). The different roles of aggrecan interaction domains. J. Histochem. Cytochem..

[12] Chen N., Gao R.F., Yuan F.L., Zhao M.D. (2016). Recombinant Human Endostatin Suppresses Mouse Osteoclast Formation by Inhibiting the NF-κB and MAPKs Signaling Pathways. Front. Pharmacol..

[13] Zhang Y., Pizzute T., Pei M. (2014). A review of crosstalk between MAPK and Wnt signals and its impact on cartilage regeneration. Cell Tissue Res..

[14] Liao J., Gu Q., Liu Z., Wang H., Yang X., Yan R., Zhang X., Song S., Wen L., Wang Y. (2024). Edge advances in nanodrug therapies for osteoarthritis treatment. Front. Pharmacol..

[15] Jiang P., Hu K., Jin L., Luo Z. (2024). A brief review of current treatment options for osteoarthritis including disease-modifying osteoarthritis drugs (DMOADs) and novel therapeutics. Ann. Med. Surg..

[16] Vakil N. (2024). Peptic Ulcer Disease: A Review. JAMA.

[17] Colletti A., Cicero A.F.G. (2021). Nutraceutical approach to chronic osteoarthritis: From molecular research to clinical evidence. Int. J. Mol. Sci..

[18] Jessberger S., Högger P., Genest F., Salter D.M., Seefried L. (2017). Cellular pharmacodynamic effects of Pycnogenol^®^ in patients with severe osteoarthritis: A randomized controlled pilot study. BMC Complement Altern. Med..

[19] Bideshki M.V., Jourabchi-Ghadim N., Radkhah N., Behzadi M., Asemani S., Jamilian P., Zarezadeh M. (2024). The efficacy of curcumin in relieving osteoarthritis: A meta-analysis of meta-analyses. Phytother. Res..

[20] Lansky E.P., Paavilainen H.M., Pawlus A.D., Newman R.A. (2008). *Ficus* spp. (fig): Ethnobotany and potential as anticancer and antiinflammatory agents. J. Ethnopharmacol..

[21] Su C., Li N., Ren R., Wang Y., Su X., Lu F., Zong R., Yang L., Ma X. (2021). Progress in the medicinal value, bioactive compounds, and pharmacological activities of *Gynostemma pentaphyllum*. Molecules.

[22] Dai N., Zhao F.F., Fang M., Pu F.L., Kong L.Y., Liu J.P. (2022). *Gynostemma pentaphyllum* for dyslipidemia: A systematic review of randomized controlled trials. Front. Pharmacol..

[23] Jiang F.Y., Yue S.R., Tan Y.Y., Tang N., Xu Y.S., Zhang B.J., Mao Y.J., Xue Z.S., Lu A.P., Liu B.C. (2024). *Gynostemma pentaphyllum* extract alleviates NASH in mice: Exploration of inflammation and gut microbiota. Nutrients.

[24] Yang K., Zhang H., Luo Y., Zhang J., Wang M., Liao P., Cao L., Guo P., Sun G., Sun X. (2017). Gypenoside XVII prevents atherosclerosis by attenuating endothelial apoptosis and oxidative stress: Insight into the ER-Mediated PI3K/Akt pathway. Int. J. Mol. Sci..

[25] Shen Z., Gao X., Huang D., Xu X., Shen J. (2024). The potential of *Gynostemma pentaphyllum* in the treatment of hyperlipidemia and its interaction with the LOX1-PI3K-AKT-eNOS pathway. Food Sci. Nutr..

[26] Guo M., Pei W.J., Liu L., Chen K., Cheng Y., Piao X.L. (2024). Neuroprotective effects of gypenosides on LPS-induced anxiety and depression-like behaviors. Int. Immunopharmacol..

[27] Pan L., Lan B., Li S., Jin Y., Cui M., Xia Y., Wei S., Huang H. (2024). Gypenoside inhibits gastric cancer proliferation by suppressing glycolysis via the Hippo pathway. Sci. Rep..

[28] Gauhar R., Hwang S.L., Jeong S.S., Kim J.E., Song H., Park D.C., Song K.S., Kim T.Y., Oh W.K., Huh T.L. (2012). Heat-processed *Gynostemma pentaphyllum* extract improves obesity in ob/ob mice by activating AMP-activated protein kinase. Biotechnol. Lett..

[29] Nguyen P.H., Gauhar R., Hwang S.L., Dao T.T., Park D.C., Kim J.E., Song H., Huh T.L., Oh W.K. (2011). New dammarane-type glucosides as potential activators of AMP-activated protein kinase (AMPK) from *Gynostemma pentaphyllum*. Bioorg. Med. Chem..

[30] Salis Z., Gallagher R., Lawler L., Sainsbury A. (2025). Loss of body weight is dose-dependently associated with reductions in symptoms of hip osteoarthritis. Int. J. Obes..

[31] Zhao X., Wang T., Li N., Meng Z., Wang W., Wang B., Song D. (2024). Potential Effects of Indomethacin on Alleviating Osteoarthritis Progression in Vitro. J. Musculoskelet. Neuronal. Interact..

[32] Maniar K.H., Jones L.A., Gopalakrishna R. (2017). Lowering side effects of NSAID usage in osteoarthritis: Recent attempts at minimizing dosage. Expert Opin. Pharmacother..

[33] Goldring M.B. (2000). Osteoarthritis and cartilage: The role of cytokines. Curr. Rheumatol. Rep..

[34] Park S.H., Huh T.L., Kim S.Y., Oh M.R., TirupathiPichiah P.B., Chae S.W., Cha Y.S. (2014). Antiobesity effect of *Gynostemma pentaphyllum* extract (actiponin): A randomized, double-blind, placebo-controlled trial. Obesity.

[35] Kim M.J., Yang Y.J., Min G.Y., Heo J.W., Son J.D., You Y.Z., Kim H.H., Kim G.S., Lee H.J., Yang J.H. (2025). Anti-inflammatory and antioxidant properties of *Camellia sinensis* L. extract as a potential therapeutic for atopic dermatitis through NF-κB pathway inhibition. Sci. Rep..

[36] Cargnello M., Roux P.P. (2001). Activation and function of the MAPKs and their substrates, the MAPK-activated protein kinases. Microbiol. Mol. Biol. Rev..

[37] Oechkinghause A., Hayden M., Ghosh S. (2011). Crosstalk in NF-kB signaling pathways. Nat. Immunol..

[38] Iijima H., Aoyama T., Ito A., Tajino J., Nagai M., Zhang X., Yamaguchi S., Akiyama H., Kuroki H. (2014). Destabilization of the medial meniscus leads to subchondral bone defects and site-specific cartilage degeneration in an experimental rat model. Osteoarthr. Cartil..

[39] Kobayashi M., Harada S., Fujimoto N., Nomura Y. (2022). Apple polyphenols exhibits chondroprotective changes of synovium and prevents knee osteoarthritis. Bioche. Biophys. res. commun..

